# Hospital and Institutional News

**Published:** 1914-04-18

**Authors:** 


### HOSPITAL AND INSTITUTIONAL NEWS.

### THE DOCTORS' WIVES ASSOCIATION.

Coupons and letters continue to reach us from doctors' wives who propose joining the suggested Association, and in thanking them for their interest and support of the proposition, we take this opportunity of adding that it is impossible for us at present to acknowledge individually their letters and enclosures. Those who have sent cheques or postal orders, as well as those who have despatched the coupon only, will, however, be communicated with individually, in due course. It is at once evident that the suggestion of the formation of a Doctors' Wives Association on the co-operative plan, for the purpose of starting a free service and purchasing agency, on the lines laid down in the article which appeared in The Hospital last month, has found a considerable degree of support; but it is incumbent on us to postpone drawing conclusions as to its immediate practicability until the time limit, April 18, has been actually reached, since, notwithstanding our weekly insistence on the need for prompt replies, letters continue to arrive from those—new readers and old—who have taken time to make up their minds as to the value of participation in the scheme. To those who have already signified their support we can only repeat the advice previously given—namely, that each and all should become centres of interest, as it is on the snowball principle that sufficient numbers will be enrolled to secure the success of the undertaking. Just as each snowflake is a potential snowball, so let each would-be member do her best to attract others, and in this way the numbers will rapidly continue to grow. The coupon appears on page 80.

### INDUSTRIAL ASSURANCE.

In our correspondence columns will be found a letter from Mr. Sidney Webb asking for information upon certain important points in connection with industrial life assurance. This new inquiry by the Fabian Research Department touches on matters which are of the greatest importance to the well-being of the labouring classes. The investigations specified should result in information of the highest value, provided they are conducted solely with an eye to the elucidation of the facts. Everyone is ready to believe that Mr. Webb and his friends are animated by good motives. But it is also well known that they hold very vehemently certain political opinions and views. It may, therefore, be necessary to regard with a critical eye the results of the research which the Fabians now propose to prosecute. We shall watch with interest the facts disclosed, and draw for ourselves the inferences.

### A CRUCIAL MOMENT AT CARDIFF.

A momentous decision was arrived at last week during the monthly meeting of the board of management of King Edward VII.'s Hospital, Cardiff, when Colonel Bruce Vaughan's motion to open the remaining fifty equipped beds was unanimously carried. In a most interesting speech Colonel Bruce Vaughan traced the hospital's astonishing growth during the past seven years, since General Lee's supplementary report appealing for £30,000 to provide the new wing was issued. Mr. John Cory's prompt offer to give £5,000 if the remaining £25,000 were raised in six months; the actual raising of this sum in nine months in contributions ranging from Mr. Cory's gift of four figures to sixpence; the latter's second challenge the following year to add 25 per cent. to every new subscription until the income of the hospital were raised to £7,000, so that additional cost of maintenance might be met; the quickness with which his example was followed, after he himself had passed away, by the addition of £98,000 to their invested capital, were all related, and form an invaluable and inspiring collection of facts which augurs well for the success of the Cardiff medical school, now at the most crucial point of its history. Cardiff had far better remain as it is than become a second-rate complete school. The only way to avoid this last is to strain every nerve to secure a Treasury grant equal to that recommended by the Royal Commission on Medical Education in the London University. The Commissioners recommended £12,000 a year to three units, and without an adequate Treasury grant Wales had better be without a complete school. Now is the time to drive this point home, for much of the splendid energy displayed in raising over a quarter million towards the founding of a complete medical school in Cardiff will yet fail of its effect if an adequate Treasury grant be not forthcoming. The inter-departmental committee, which was formed after Mr. D. Lloyd George had received a deputation, will be sitting next week to determine the amount of the grant which is to be given. The Treasury has a great opportunity for founding a work whose benefits to mankind cannot fail to be out of all proportion to the money expended.

### MATERNITY BEDS TO BE OPENED.

Col. Bruce Vaughan pointed out that few other hospitals, in fact, had such a fine reserve fund. It amounts to-day to £140,000, and the history of the new wing more than justified the opening of the additional beds. He urged the allocation of five beds to maternity cases, for which, he said, there was no provision in the city so far as respectable women were concerned. They were also essential for the education of the senior medical students. The truth, in fact, is that these beds are necessary to provide the statutory number required by the General Medical Council for a complete medical school, while the present waiting list of 700 patients provides the popular driving force to this argument. Indeed, perhaps the most poignant criticism that could be made of the voluntary hospitals generally to-day is the inadequacy of their accommodation to the need of the population.

### THE HUMAN SIDE OF HOSPITAL FINANCE.

But there is another side to this wonderful development of hospital activity in Cardiff. A great voluntary hospital does not become great without infusing something of this quality into the lives of those, who work for it and the patients whom it serves. What is the human aspect of this income, growth of bricks and mortar and bank investments? Without Colonel Bruce Vaughan this progress would hardly have been possible. But as the voluntary system of personal service for the sick makes the men who live up to its ideal in very truth "members one of another," we see here how the man of leisure, the man of money, and the paid administrator who has consecrated his life to a highly skilled profession, combine together to achieve this prodigy of growth. Colonel Bruce Vaughan, General Lee, with Sir W. J. Thomas and Mr. Leonard Rea, are among the leading spirits to whom the inspiration and direction, the provision of ways and means, and the executive work have been entrusted. All have risen to the level of the opportunity offered to them, and we note, with a satisfaction that all hospital secretaries will share, that Mr. L. D. Rea's salary has been raised to £450, rising by five annual increments to £500. Since the income of the hospital is now in round figures £16,000, this means that Mr. Rea is being paid at the rate of 3 per cent. on the income of his institution, a rate which our experience has taught us to regard as a basis for chief administrative salaries. Were hospital finance and administration a purely commercial concern, the manager, as the secretary would probably be called, of a business turning over £16,000 a year would certainly be paid at this figure, and quite possibly at a higher one. But, though the business side of a secretary's work has increased enormously during even the past ten years, the hospital secretary still requires more varied and skilled qualities than are necessary to the management of most "business houses." The work, to that extent, remains its own reward, and rightly so. For what first-class man would exchange appeal-drafting for profit-hunting?

### THE HOSPITAL SECRETARY'S HOUSE.

Moreover, the secretary of a great institution differs from the average business man in that his office (meaning his institution) is never closed, whilst even daily newspapers close on Saturdays, having no Sunday issue to prepare for. The result is that practically all his time is spent in the institution, and in some instances, as at Derby, the secretary is provided with a house in the grounds, and with the emoluments of fuel and light. What effect does this arrangement have on a secretary's salary? Probably opinions will differ, but, broadly speaking, emoluments are more valuable than money. Though any man who is allowed, say, £60 a year in lieu of a house, can probably secure a roof over his head at this rental, it is yet generally true that the house provided for him (if one there be) will be a better and more convenient one than he could procure at this figure. To receive an emolument is like buying wholesale. It costs a large firm, organisation, or institution less to feed and house any officer than it would cost himself. It, therefore, pays both officer and organisation to house and feed him better than to raise his salary. It pays the man directly, and the employer indirectly, because, for any given sum that the employer is prepared to pay, he can give in kind to his officer a more than equivalent prestige and comfort, and thereby make him more efficient. Is not the size of a man's house still more regarded as a test of position than actual income or occupation by themselves? The case against the secretary's house, as more obvious, needs less emphasis here, but we shall be ready to publish letters on the subject.

### A LESSON FROM ULSTER AT WORTHING.

A public demonstration has been given by the Worthing Detachment of the British Red Cross Society of the way in which a building can be converted into a field hospital in time of war, and the Sussex Road Girls' School was chosen for the experiment. The hall and class-rooms were converted into wards, stores departments, and kitchen, and an operating theatre was also equipped. The beds used were generally of the camp variety, but in one "ward" improvised beds of straw were used. The detachment possesses a camp cooking stove, but a demonstration was given of the method of cooking with oil-stoves and—after the manner of expert campers—with old biscuit-tins as ovens. The "cases" demonstrated included "abdominal wounds," "fractures," "pneumonia," and "enteric." Perhaps the present condition of emergency hospital work in Ulster may have one feature of lasting good which will outlive the present anxiety—that, namely, of encouraging practical ambulance, Red Cross, and auxiliary hospital work throughout the United Kingdom.

### SALARIES AND THE SATURDAY FUND.

At the quarterly meeting of the Board of Delegates of the Hospital Saturday Fund, Sir Thomas Vezey Strong, Bart., presided for the first time since his election as chairman of the governing body. There were 124 representatives present. The report of the Executive Committee *re* salaries and wages of the official staff, which was referred to in The Hospital of January 10, was considered in private. At the close of the meeting it was stated by the Secretary, Mr. A. W. Davis, that the report had been generally adopted, with the exception that most of the weekly-paid servants would receive increases beyond those recommended by the Executive Committee. At the commencement of the meeting the Secretary announced that since the last sitting eighty additional certificates of membership had been received. Twenty-seven were from representatives of contributing firms; thirteen from local committees; and forty from participating institutions. The Fund to date—£3,556—exceeded that of last year at the same period by £476.

### THE TREATMENT OF UNINSURED TUBERCULOUS PATIENTS.

The Metropolitan Asylums Board, which has been considering a report from the General Purposes Committee on the treatment of uninsured tuberculosis patients, with reference to whom a correspondence has taken place between the Board and the London County Council, has resolved to place the Committee's conclusions before the Local Government Board. The Committee's proposal, in brief, is that the Managers should deal with these cases under Section 80 of the Public Health (London) Act, 1891. If the Committee's proposal be acted upon, instead of the Council raising half the cost this half would be raised by the Board in the same manner as is now the case concerning the fever and diphtheria patients. At present the Government grant and the county rates bear the cost between them.

### ALTERING THE RULES AT GRAVESEND HOSPITAL.

Alderman A. E. Enfield, Mayor of Gravesend, in moving the adoption of the report of the committee of management and treasurer's accounts of the Gravesend Hospital at the annual meeting of the governors last week, remarked that the revenue account showed a credit balance of £750, when this same account, as pointed out by the Treasurer, was, as a rule, £400 behind. The report, therefore, was received with genuine warmth. Mr. Byrne, Mr. Davis, Mr. Porter, Mr. Wilson Rose, and Mr. Trebble were re-elected members of the committee of management, and Mr. J. H. Cooper, jun., was elected to the vacancy caused by the promotion of Mr. John Russell as a vice-president. It is noteworthy that new rules were submitted and adopted in place, we understand, of all existing rules—a feat which can rarely be accomplished by institutional governors without a degree of warmth of a less genial kind.

### SUMMER SEVERITIES AT SOUTHWARK INFIRMARY.

Nine months have elapsed since the Local Government Board called upon the Camberwell Borough Council to remove the dust-siding at East Dulwich Station, close to the Southwark Infirmary, but the request has so far been ignored. Last summer, to recall once more, there was a serious outbreak of enteritis amongst the children in the infirmary, resulting in nine deaths, which Dr. Bruce, the medical superintendent, attributed to the nuisance arising from the dust-siding. The Local Government Board's request was met by the Borough Council producing their officials' reports on the siding, which claimed that the nuisance was purely an æsthetic one. Thereupon the Local Government Board addressed a letter to the Council stating that they "could not accept the conclusion that the nuisance to the adjacent infirmary is purely an æsthetic one." They were satisfied that the condition complained of did at times give rise to a real nuisance, and they were still of opinion that other or improved arrangements should be made by the Council for dealing with the refuse. The Council has decided merely to acknowledge the letter, thereby implying that they do not intend to take action. With the near approach of summer, and a probable repetition of the nuisance in its aggravated form, action should be immediately taken by the Southwark Board of Guardians to get the dust-siding closed, if, indeed, the Local Government Board does not forestall them in doing so. Dr. Bruce has done all that is possible for him to do on behalf of the children committed to his care, and now is the time for those in authority to move quickly if the infirmary is not to continue a series of reports recording in all seriousness that summer has set in with its usual severity in Southwark.

### INFIRMARIES AND THE INSURANCE ACT.

The Epsom Guardians have been making inquiries into the number of insured persons admitted to their workhouse infirmary. They find that out of a total of 173 adult patients admitted from October 13, 1913, to February 28, 1914, fifty-five were insured persons. Their experience tallies with that of most Boards of Guardians. A census taken the other day in a London infirmary, for instance, showed that out of 355 adult inmates eighty-eight were insured. The Epsom Guardians have sent a letter to the Local Government Board pointing out that under the National Insurance Act an insured person treated in a Poor-Law infirmary must receive from his approved society after his discharge the sickness benefit which has accumulated, unless it has been expended in making provision for his dependants, or on surgical appliances, or otherwise for his benefit. The Guardians suggest the Act should be amended so as to empower them to make arrangements with the approved society for the benefit to be paid to them whilst an insured person is an inmate of their institutions. They also suggest that whilst the insured person is in the infirmary the money payable to the panel doctor should be deducted from him and paid to the infirmary medical officer. Whilst we admit that the power a panel doctor possesses of sending his insured patients to an institution may be abused, it is so often impossible to carry out satisfactorily the treatment of serious diseases in the homes of the insured that it would not seem desirable to penalise him for obtaining institutional treatment for his insured patients.

### A MEDICAL REVOLUTION IN MADEIRA.

A report has gained currency that the Portuguese Administrator of Funchal, the capital of Madeira, has intimated to the English and American doctors that they must either cease practice or produce a recent diploma from a Portuguese University. The Administrator is reported to have said to those who could show diplomas of some years back: "We are not responsible for the doings of the Monarchy. By the law of the Republic you must all have Portuguese diplomas or go." We can hardly credit the possibility of such official action even in a country governed by the Portuguese, since, if the English doctors are compelled to leave, most of the foreign colony will leave with them. The very introduction of trained nurses into Madeira was due to English enterprise.

### VOLUNTARY VERSUS MUNICIPAL DISPENSARIES.

The Bermondsey Borough Council, anxious to establish a municipal dispensary, have asked the Local Government Board to sanction a municipal scheme paid for by the Council for the home treatment of uninsured persons, involving an annual expenditure of £1,000. They also asked whether the Board would contribute four-fifths for capital expenditure on buildings, and whether they would contribute 50 per cent. of the upkeep if they approved of the scheme. The Board have informed the Council that before they establish a municipal dispensary they should first of all ascertain if the needs of the Borough could be met by utilising the services of Guy's Hospital and the existing voluntary dispensary. The voluntary dispensary is to be moved to fresh premises in the centre of the Borough, where a fully-equipped dispensary would be at the service of the Council if they determine to avail themselves of it. The committee desire the Council to make an annual grant of £200 towards their funds. Unfortunately, a certain amount of party feeling has led a section of the Council to urge their colleagues to favour an expensive municipal dispensary. In the meantime nothing is done to further either one scheme or the other. One point which has converted many councillors to co-opt with the voluntary dispensary is that the medical staff there are able to visit patients in their own homes, if they are unable to visit the dispensary, without any of the trouble of applying to the Local Government Board for permission.

### DOCTOR AS PRISONS COMMISSIONER.

The King has appointed Sir Herbert Smalley, M.D., one of the Commissioners under the Prisons Act of 1877. Sir Herbert, who took the Conjoint Diploma in 1875, was educated at King's College, and eventually became an M.D. of Durham University. Since 1897 he has held the post of Medical Inspector of Prisons and Superintendent Officer of Convict Local Prisons. He has always taken a keen interest in the hospital problems of prison administration, of which the hunger-striking militant women suffragists have advertised one so well of late, and has published a work on "Prison Hospital Nursing."

### A HOVE PUBLIC MEDICAL SERVICE.

A Public Medical Service has been organised for Brighton, Preston, Hove, and district for those people who, whilst not insured, are unable to pay the ordinary fees for medical attendance. The income limit has been fixed at two pounds per week, the income of the whole family being included in this amount. Some forty medical men, we understand, have already joined the Service and have undertaken not to attend any patients, whether club or otherwise, at a lower rate than that fixed by the Service—namely, 8s. 8d. per annum for adults, and 4s. 4d. per annum for children. The affairs of the Service are managed by a committee of medical men, among whom are several well-known practitioners in the district, who, although not taking any active part in the actual practice of the Service, are still helping their colleagues with advice and assistance in the management. Several of them have loaned sums of money to the Service to assist in the payment of the initial expenses, the loans to be later repaid out of the receipts from the patients. It seems that up to the present about six hundred of the public have joined, and a paid collector gathers in their contributions. To make the Service a success, it is estimated that probably at least 3,000 are needed, and it is to be hoped that before long the numbers will be largely added to. The Service is receiving the assistance and support of the clergy and others interested in social work. The committee of the Service hope that in the event of sufficient support they will be able to extend the benefits by additional advantages, *e.g.* nursing, loan of sick appliances, etc., for those patients who may require them.

### DOCTOR'S PRECAUTION AGAINST PREMATURE BURIAL.

Dr. William Clunie Wise, M.D., D.P.H., of Ealing, W., who was also a barrister-at-law, and who died on February 7, aged seventy-six, left estate of the gross value of £9,727. He expressed the wish that the coffin in which his body should lie should not be closed down for five days after his supposed death and that his funeral should not take place until seven days after his death. Would not cremation have been a simpler way out of the difficulty?

### CHARLES DICKENS ON WARD WORK.

No doubt just because he divined the soul of Mrs. Gamp so penetratingly Charles Dickens was a warm testifier to nursing improvements, and, as a correspondent reminds us, it was while he was actively conducting *Household Words* that the following passage appeared in one of its numbers: "I often think, when I walk through the long, clean, silent wards of an hospital (nothing, save the lower decks of a man-of-war, can come up to hospital order, neatness, and cleanliness), watching the patients quietly resigned, yet so expressively suffering, the golden sunlight playing on their wan faces, the slow crawling steps of the convalescents, the intermittent cases sitting quietly at their beds' foot waiting patiently till their time of endurance shall come, hearing the monotonous ticking of the clock, the slow rustling of the bedclothes, the pattering foot of the nurse as she moves from bed to bed, consulting the paper at the bed-head as to the medicine and diet, and slowly gurgling forth the draught. I often think what an immense, an awful weight of responsibility hangs in this abode upon the nurse. The doctor has his vocation, and performs it. He severs this diseased limb and binds up that wound. The physician points out the path to health, and gives us drugs like money to help us on our way. But it is for the nurse to soothe and tend and minister to him until the incubus of sickness be taken off, and he struggle into life a whole man again." What a picture of the sensitive lay visitor's impressions of a hospital ward nearly half a century ago! While institutional workers can naturally adjust the perspective here and there, let all remember that to give such an impression as the above to the layman must be the aim, as it is the guarantee, of the voluntary hospital system. It is the atmosphere which patients expect, and it is certainly one which no State hospitals can give them.

### THE CHAPLAIN'S DEPARTMENT.

On another page of this issue appears a remarkable article by a clergyman on "Some Hospital Chapels." The interest of its historical survey is enhanced by the illustrations which accompany the text, and we believe that institutional readers with a turn for history will appreciate it as much as the series of articles on the "Origin and Evolution of the Eighteenth Century Hospital Movement" which came to an end in last week's issue. For the latter we have received several letters of thanks and appreciation from readers in many parts of the United Kingdom, to whom, therefore, we specially commend the article on "Some Hospital Chapels" already referred to. It is interesting in this connection to note that one of our March Questions in the *Institutional Worker* Supplement dealt with the Chaplain's Department, and asked, "What system should be employed in an institution served by an honorary non-resident chaplain in order (1) to secure efficient visitation; and (2) to avoid any friction, or to hinder routine work?" We are glad to state that among the interesting responses that we have received to this Question is a remarkable one from East Anglia, where the system in practice at the Norfolk and Norwich Hospital is described in detail. We hope to publish this reply next week, and suggest that the article on "Some Hospital Chapels" and the forthcoming account of the chaplain's department at Norwich will materially illuminate side aspects of each other.

### THE CICELY NORTHCOTE TRUST.

The valuable work done by this endowment is well illustrated by the fourth annual report, lately published. The Trust was established by Mr. H. H. Stafford Northcote, who provided an endowment fund of £15,000; but subscriptions are also required to carry on the work which the Trust does, and, indeed, the income is insufficient to meet all the calls which the trustees would like to respond to. The Trust is established in connection with St. Thomas' Hospital for the purpose of "providing such sympathetic assistance to patients as by relieving their minds shall enable them to profit fully by professional treatment given in the hospital." In 1913, it appears, some 6,440 patients in the wards were approached; and as a result of this investigation 1,094 homes were visited and helped in various ways; while 1,000 patients were referred either to the Charity Organisation Society, the parish clergy, or the Invalid Children's Aid Association. St. Thomas' Hospital is fortunate in having so splendidly endowed an organisation for this most excellent work; and it were to be wished that all large hospitals could have similar departments founded and kept going. This kind of help is, perforce, neglected at most hospitals for want of funds; yet the anxieties of the breadwinner for the welfare of his dependants while he is in hospital often militate against the full success of his treatment. Nor is this the only sphere of the Trust's activities, a complete account of which in the report makes fascinating reading. The organisation seems to be very thorough and effective, and the whole experiment a great success.

### DOCTOR, PULPIT AND PATIENT.

We are indebted to an ex-hospital chaplain for an account of the following curious circumstance: "The Rev. S. Shrubsole, a Congregational minister, was advertised to occupy the pulpit on April 5 at Cheltenham (he writes), but unfortunately fell ill with a sharp attack of influenza, whereupon his medical adviser, Dr. Hutton, refused permission for his patient to officiate. It was too late to obtain another minister to fill the gap thus caused, but Dr. Hutton stepped into the breach and preached on his patient's behalf, and, it is said, delivered a sermon upon the subject that had been already advertised." It is not every medical man who would have the courage to do this, but perhaps in consenting he remembered that the patient could never take his own place in the operating theatre, however valuable the minister's aid might be afterwards in the ward.

### A RADIUM INSTITUTE FOR GLASGOW.

We were able to state a few weeks ago (The Hospital, February 21, 1914, p. 554) that a political pamphlet was being sold in Glasgow, the profits from which were to help in buying radium; and other means have been under consideration for procuring radium on behalf of the various voluntary institutions in Glasgow which could make use of this new therapeutic agent. It is now announced that definite steps have been taken for the formation of a Central Radium Institute in that city, which has thus followed the example lately set by Manchester. A committee is being formed, and a public appeal will be made for about £9,000, wherewith some three hundred milligrammes of radium will be purchased and the Central Institute set on foot. It is intended that the radium shall be available for hospitals and, under certain conditions, for the patients of private practitioners. Thus the Glasgow (and Manchester) type of Radium Institute differs from that which has been so successfully started in London in that the latter does not lend any of its stock of radium to hospitals, though it treats the bulk of its patients on charitable lines and does not exist for profit. Probably each type is suited to the local conditions: London is the only city large enough to support a Radium Institute of this latter type, whereas in Manchester and Glasgow, large and wealthy as they are, the modified system which they have adopted is the more advantageous. It is not stated whether the sale of the pamphlet referred to (in support of the Home Rule Bill) has brought in anything towards the £9,000 required.

### THE BLACKPOOL HOSPITAL DEADLOCK.

The adjourned annual meeting of the donors and subscribers to the Victoria Hospital, Blackpool, was held last week, and occupied three hours of heated discussion. The ostensible business for which it was called was to elect three additional elective members of the board of management, thus to increase the number from twelve to fifteen in accordance with the special resolution passed at the recent annual meeting. An attempt was made by the Rev. A. W. R. Little to draw some explanation of the present position from the chairman, Alderman J. Bickerstaffe, who stated that the latest resignations of nurses and wardmaids had been made in loyalty to the matron. The chairman added that the local medical men had been most unreasonable in demanding resignations of other officials before consenting to meet the board. Mr. W. J. Read blamed the board for their unskilful handling of the long dispute, which he traced in detail, from the outset. Alderman Heap replied, but the only speaker who really impressed his audience, and voiced the latent common-sense view of the meeting, was the Rev. S. Gamble-Walker, who reminded the disputants that neither the medical men nor the board were masters of the situation, but the subscribing public. He characterised the present *impasse* as a scandal to the county.

### THE DUTY OF THE SUBSCRIBERS.

The net result was merely the passing of two tame resolutions, which gave power to the board to induce the doctors to come on to the medical board, and if they absolutely refused, and still adhered to their conditions, to empower the board to use their judgment in securing a suitable medical staff to work the hospital. How this can be done in view of the united opposition of the local profession is not apparent. The new attempt is merely prolongation of the quarrel. In other words, the issue was talked out, and we can only suggest that the more responsible men among the subscribers should call a meeting, agree that an end must be immediately put to the present disgraceful squabble, and, if necessary, call on the existing board of management to resign *en bloc*, so that a clean start may be made for a new administration, which would appoint its medical officers in the ordinary way as if a new hospital were being opened. Who will follow the lead given by Mr. Gamble-Walker? That such a policy would command the confidence of the public the impression made by his speech at last week's meeting is sufficient to show.

### HOSPITALS AND SOCIAL FUNCTIONS.

Next Wednesday the reconstructed children's hospital in connection with the Leicester Royal Infirmary will be opened by the Hon. Mrs. G. Murray Smith. As is usual at this hospital's functions, a distinguished and, what is more, an enthusiastic audience is expected to be present at the ceremony, on the invitation of Sir Edward Wood, the chairman, and the board of governors. There are few better tests of a voluntary hospital's efficient administration, in the broadest sense of its close as well as scientific relation to the community which it serves, than such ceremonies as this. At Leicester these events are given all the distinction of a social function, while preserving the air of importance which the participation in public work alone can give. The corner-stones of the voluntary system, that it exists to uphold, are personal responsibility and personal service, which, on occasions like these, it is its duty to teach, not disdaining the ritual of spring sunshine, gay dresses, and holiday spirits, but letting all these be the expression of that true gaiety of heart which is the real reward of unselfish work in the world.

### LORD ST. ALDWYN ON THE VOLUNTARY SYSTEM.

In some remarks in this column last week we discussed the best way of enlivening the annual meeting, and pointed out that the meetings which are most successful are not those which rely on extraneous aids, like cinema slides, processions, or social personages, as an analgesic to the business in hand, but those which deliberately give that business the very first place, and lend to it all the emphasis of well-informed hospital authorities as speakers. To the examples which were quoted of the right way to save the annual meeting from perishing of *ennui* without degrading it, may be added that of the Cheltenham General Hospital, over which Lord St. Aldwyn presided recently. He took occasion to defend the voluntary system as a stimulus to the national character, and warned his hearers to put aside all thought of their hospital being handed over to municipal management. Institutions wise enough to secure important, as opposed to society, public speakers will not only interest their governors but secure a wide advertisement in the public press.

### THIS WEEK'S DRUG MARKET.

Business has been at a standstill during the greater part of the week owing to the Easter holidays, and few price changes of any note have taken place. The advanced price of citric is well maintained, and there seems to be no immediate prospect of any decline, as the difficulty of obtaining supplies has become more pronounced; there has been a further advance in quotations for potassium citrate, sodium citrate, and iron and ammonium citrate. There is little change in the position of cod-liver oil, and buyers seem more disposed to come forward; the present prices may be regarded as comparatively low, and those who are in need of supplies would perhaps not be ill-advised to make their purchases without much further delay. Belladonna root continues scarce and dear, with little prospect of lower prices at present. English pressed almond oil is slightly lower in price. Opium is unchanged.

